# Association between Breastfeeding and DNA Methylation over the Life Course: Findings from the Avon Longitudinal Study of Parents and Children (ALSPAC)

**DOI:** 10.3390/nu12113309

**Published:** 2020-10-29

**Authors:** Fernando Pires Hartwig, George Davey Smith, Andrew J. Simpkin, Cesar Gomes Victora, Caroline L. Relton, Doretta Caramaschi

**Affiliations:** 1 Postgraduate Programme in Epidemiology, Department of Social Medicine, Federal University of Pelotas, Pelotas 96020-220, Brazil; cvictora@gmail.com; 2 MRC Integrative Epidemiology Unit, Population Health Science, Bristol Medical School, University of Bristol, Bristol BS8 2BN, UK; kz.davey-smith@bristol.ac.uk (G.D.S.); andrew.simpkin@bristol.ac.uk (A.J.S.); caroline.relton@bristol.ac.uk (C.L.R.); d.caramaschi@bristol.ac.uk (D.C.); 3 School of Mathematics, Statistics and Applied Mathematics, National University of Ireland, H91 TK33 Galway, Ireland

**Keywords:** breastfeeding, life-course, DNA methylation, epigenome-wide association study

## Abstract

Background: Breastfeeding is associated with short and long-term health benefits. Long-term effects might be mediated by epigenetic mechanisms, yet the literature on this topic is scarce. We performed the first epigenome-wide association study of infant feeding, comparing breastfed vs non-breastfed children. We measured DNA methylation in children from peripheral blood collected in childhood (age 7 years, N = 640) and adolescence (age 15–17 years, N = 709) within the Accessible Resource for Integrated Epigenomic Studies (ARIES) project, part of the larger Avon Longitudinal Study of Parents and Children (ALSPAC) cohort. Cord blood methylation (N = 702) was used as a negative control for potential pre-natal residual confounding. Results: Two differentially-methylated sites presented directionally-consistent associations with breastfeeding at ages 7 and 15–17 years, but not at birth. Twelve differentially-methylated regions in relation to breastfeeding were identified, and for three of them there was evidence of directional concordance between ages 7 and 15–17 years, but not between birth and age 7 years. Conclusions: Our findings indicate that DNA methylation in childhood and adolescence may be predicted by breastfeeding, but further studies with sufficiently large samples for replication are required to identify robust associations.

## 1. Background

Breastfeeding has clear short-term health benefits, particularly in reducing the risk of infections in childhood. Accumulating evidence indicates that breastfeeding may also have long-term effects on health outcomes and human capital, as well as benefit maternal health [1]. Being breastfed has been associated with lower risk of being overweight or obese and having type 2 diabetes in adulthood in the most recent meta-analysis based on a systematic literature review [2]. Moreover, it has been suggested that being breastfed has a positive effect on cognitive development in studies using complementary epidemiological approaches [3,4,5]. However, it should be noted that the single large randomized controlled trial of breastfeeding promotion did not support a causal effect of breastfeeding on obesity in childhood and adolescence [6,7], and on metabolic processes related to type 2 diabetes (such as blood glucose and insulin) in childhood [8]. The same trial indicated that the effect of breastfeeding on cognitive development in childhood is attenuated over time [4,9]. However, a single trial does not fully dismiss many high-quality observational studies (especially those conducted in different contexts from that of the trial and/or when using different designs—and, therefore, prone to different potential bias sources—yield similar conclusions). In summary, definitive conclusions on the long-term effects of breastfeeding cannot be drawn from the current epidemiological evidence.

The mechanisms underlying the putative long-term effects of breastfeeding are not fully understood. Such mechanisms clearly must persist over time after weaning—in other words, become embodied in the organism [10]. In the case of other early-life exposures such as maternal smoking during pregnancy, there is evidence of long-term associations with offspring DNA methylation [11]—i.e., addition of a methyl (–CH3) group to DNA at the 5′ position of a cytosine base, typically in cytosine-guanine (CpG) dinucleotides [12]. DNA methylation is one type of a broader class of biological processes known as epigenetics, which encompasses mitotically heritable events—other than changes in the DNA sequence itself—involved in gene expression regulation. Epigenetic processes play a key role in developmental processes [13,14], and have more recently been linked to disease processes [15,16,17], although causal claims have been overstated in many cases [18,19].

Some evidence suggests that breastfeeding might influence DNA methylation through the effects of some of its nutritional components [20] or through the microbiome, which is shaped by early feeding habits [21]. However, according to a systematic literature review [22] the overall evidence on the potential effects of breastfeeding on DNA methylation is scarce and reports of differences in DNA methylation according to breastfeeding are only emerging in the literature [23,24,25,26,27]. Our aim was to perform a genome-wide assessment of the association between breastfeeding and DNA methylation in childhood, characterize—if present—the pattern of this association and investigate whether it persists until adolescence in a population-based study in England.

## 2. Methods

### 2.1. Study Setting and Participants

Study subjects were part of the Accessible Resource for Integrated Epigenomic Studies (ARIES) [28], a sub-sample of the Avon Longitudinal Study of Parents and Children (ALSPAC) for which methylation data were collected. ALSPAC is a population-based, prospective birth cohort of women and their children [29,30,31]. All pregnant women living in the geographical area of Avon (UK) with expected delivery date between 1 April 1991 and 31 December 1992 were invited to participate. Approximately 85% of the eligible population was enrolled, totalling 14,541 pregnant women who gave informed and written consent. Information on the data collection and availability can be found at http://www.bris.ac.uk/alspac/researchers/data-access/data-dictionary/. Ethical approval for the study was obtained from the ALSPAC Ethics and Law Committee and the Local Research Ethics Committees.

Our analysis was focused on the offspring born between 1991 and 1992. The analyses were restricted to singletons or only to one participant out of a twin pair (N = 2 twin pairs), selected at random. Individuals with missing information for the exposure, outcome or covariates (described below) were excluded.

Individual-level data were obtained from the ALSPAC study according to the ALSPAC data access procedures and policies detailed at http://www.bristol.ac.uk/alspac/researchers/access/.

### 2.2. Study Variables

#### 2.2.1. DNA Methylation

DNA methylation in white blood cells was measured in ARIES offspring at three time points: at birth (cord blood), and at 7 and 15–17 years of age (peripheral blood). DNA samples underwent bisulphite conversion using the Zymo EZ DNA methylation^TM^ kit (Zymo, Irvine, CA, USA). The Illumina HumanMethylation450 BeadChip was used for genome-wide epigenotyping. The arrays were scanned using an Illumina iScan, and initial quality checks performed using GenomeStudio (Illumina, Inc., 5200 Illumina Way, San Diego, CA 92122, USA) version 2011.1. A total of 71 samples across all the time-points were analysed in duplicates to ensure the technical validity of the arrays. We excluded single nucleotide polymorphisms, probes with a high detection *p*-value (i.e., *p*-value > 0.05 in more than 5% samples) and sex chromosomes. Methylation data normalisation was carried out using the “Tost” algorithm to minimise non-biological between-probe differences [32], as implemented in the “watermelon” R package [33]. All processing steps used the “meffil” R package [34].

The outcome variables of this study were cord and peripheral blood (ages 7 and 15) DNA methylation levels in ~470,000 CpG sites. Methylation was analysed as beta values, which vary from 0 to 1 and indicate the proportion of cells methylated at a particular CpG [35]. Regression coefficients and standard errors were multiplied by 100, so that they can be interpreted as percent point differences in average DNA methylation at a given CpG site.

#### 2.2.2. Breastfeeding

Breastfeeding data were collected through questionnaires answered by the mothers when their offspring were (on average) four weeks, six months and 15 months old, and combined into four different breastfeeding categorisations:

- A binary indicator of whether the individual was ever breasted (regardless of duration).
- Breastfeeding duration groups, defined as follows: 0 = never breastfed; 1 = 1 day to 3 months of duration; 2 = 3.01 to 6 months; 3 = 6.01 to 12 months; and 4 = more than 12 months.
- Same as the above but coding each category as a number and treating this as a continuous variable, thus assuming a linear trend per unit increase in duration category.
- Breastfeeding duration in months, as a continuous variable, thus assuming a linear trend per month increase in breastfeeding duration.

#### 2.2.3. Covariates

Covariates were selected mostly based on a conceptual model encoded in the form of a directed acyclic graph (DAG) that we defined previously [27]. The following covariates were used:

- Sociodemographic: an indicator of whether the participant had white ethnic background (informed by mothers at 32 weeks of gestation), and the top two ancestry-informative principal components estimated using the participant’s genome-wide genotyping data [36].
- Family socioeconomic position: to avoid collinearity issues, we used only the mother’s highest educational qualification (informed by the mothers themselves at 32 weeks of gestation).
- Maternal characteristics: parity (informed by the mothers at 18 weeks of gestation), height, pre-pregnancy weight (informed by the mothers themselves at 12 weeks of gestation), age at birth (calculated from mother’s date of birth and date of delivery) and folic acid supplementation (informed by the mothers at 18 and 32 weeks of gestation).
- Gestational characteristics: maternal smoking during pregnancy (informed by the mothers at 18 weeks of gestation), type of delivery (informed by the mothers when their offspring were eight weeks old), gestational age (calculated from the date of the mother’s last menstrual period reported at enrolment; when the mother was uncertain of this or when it conflicted with clinical assessment, the ultrasound assessment was used; where maternal report and ultrasound assessment conflicted, an experienced obstetrician reviewed clinical records and provided an estimate) and birthweight (from obstetric data, measures from the ALSPAC team and notifications or clinical records).

Although not included in the DAG, participant’s sex and age at blood collection were also selected as covariates. Given that they are associated with DNA methylation but are not influenced by breastfeeding, adjusting for those two covariates may improve power by reducing variance in DNA methylation. We also adjusted for estimated cell counts using Bakulski’s [37] (for cord blood) or Houseman’s (for peripheral blood) [38] methods to account for methylation differences due to cell composition. Finally, a surrogate variable analysis was performed on the methylation data using the “sva” R package. For each timepoint 10 surrogate variables were created and those not associated with breastfeeding were additionally included as covariates to adjust for batch effects [39].

#### 2.2.4. Statistical Analyses

We conducted an epigenome-wide association study (EWAS) of any reported breastfeeding (including mixed breast- and formula-feeding and in combination with other foods). The main EWAS analyses considered breastfeeding as the exposure in two categorisations: (i) none vs. any; (ii) duration categories, assuming a linear trend.

The outcome was DNA methylation measured at ~470,000 CpG sites in peripheral blood at the age of 7 years. The analyses were performed on the subjects with complete covariate data available (N = 702) adjusting for estimated cell composition, batch effects, and all other covariates. In all models we measured the association between breastfeeding and DNA methylation at each CpG using robust linear regression models (“rlm” function in the “MASS” R package) and tested the association by computing heteroskedasticity-consistent standard errors (“coeftest” function in the “lmtest” R package with the variance-covariance option vcovHC, type = “HC0”, from the “sandwich” R package). The association of methylation at CpGs at age 7 and breastfeeding was evaluated by looking at *p*-values corrected for the false-discovery rate (FDR) using the Benjamini and Hochberg method [40]. Associations with at least suggestive evidence, here defined as achieving a nominal *p*-value < 5.0 × 10^−6^, were further analysed using breastfeeding duration variable as exposure (either 4 categories or continuous duration).

Sensitivity analyses were further conducted to explore a potential dose-response relationship in the association of breastfeeding with DNA methylation at the suggestive sites at age 7. Breastfeeding duration during the first 3 months was categorised by 2-week increments (exposure) compared to the reference (no breastfeeding) and DNA methylation at age 7 at the suggestive sites (outcome). A further categorisation considered mixed breast-/formula feeding and exclusive breastfeeding in the first 3 months of age, compared with no breastfeeding. As the main source of food in the first 3 months of age is milk, these categories can be described approximately as “No breastfeeding”, “Mixed breast-/formula” and “Exclusive breastfeeding”. To further test the strength of the association between breastfeeding and methylation, conditional on lipid profiles, we ran the analysis on the suggestive sites with further adjustment for serum lipid profiles in the mothers and the children, separately. The lipid profiles consisted of serum total cholesterol, total cholesterol in very-low-density lipoproteins, total cholesterol in low-density lipoproteins, total cholesterol in high-density lipoproteins and serum total triglycerides. All the lipid fractions were measured using the Nightingale Health NMR-based blood biomarker analysis platform [41] in blood collected from the mothers during pregnancy and from the children at age 7. Lipid concentrations were expressed in mmol/L. To rule out a potential confounding effect of gestational methylation age we computed a gestational age score based on DNA methylation in cord blood using a published algorithm in Bohlin et al. [42] which was previously applied to ALSPAC data and found to correlate well with measured gestational age (r = 0.65) [43].

At the CpGs with suggestive evidence we also investigated whether the signal persisted over time by analysing the association of CpG methylation at age 15–17 and breastfeeding (ever vs never and duration categories). Cord blood methylation was analysed as a negative control [44], under the assumption that at least some of possible pre-natal residual confounding would result in associations between breastfeeding and cord blood methylation.

All follow-up analyses were performed using robust linear regression and heteroskedasticity-consistent standard errors (implemented as described above).

The EWAS results were further used to identify differentially methylated regions (DMRs) in relation to breastfeeding. DMRs were identified using the Comb-P method, which tags regions enriched for low *p*-values while accounting for auto-correlation and multiple testing [45,46]. Following the criteria used by Sharp et al. [47], a region was classified as a DMR if: (i) it contained at least two CpGs; (ii) all CpGs in the region are within 1000 bp of at least another CpG in the same region; and (iii) the auto-correlation and multiple-testing corrected (upon applying Stouffer-Liptak-Kechris [48] and Sidak methods [49], respectively) *p*-value for the region was <0.05. The CpGs belonging to the identified DMRs were analysed further to assess if breastfeeding had a consistent effect across the DMR (i.e., if CpGs in the DMR generally presented greater or lower levels of methylation according to breastfeeding) using linear mixed models to account for the correlation between CpGs assuming that they are nested within individuals. Therefore, each CpG in a given DMR was treated as a repeated measure of DNA methylation, and the regression coefficient indicates the average difference in DNA methylation levels comparing breastfed and never breastfed individuals, averaging across all CpGs in the DMR. This was implemented using the “nlme” R package. This was complemented by evaluating, for each DMR, the directional consistency of each CpG across time points using a sign test. Analyses were performed using R 3.4 (R Core Team (2018). R: A language and environment for statistical computing. R Foundation for Statistical Computing, Vienna, Austria https://www.R-project.org). The scripts used are available on request to the study authors.

## 3. Results

### 3.1. Description of Study Participants

Table 1 and Table S1 display the characteristics of the study participants with DNA methylation data at age 7 (N = 702) and non-missing information for all study variables (corresponding to approximately 70% of all ARIES participants). In general, the subset included in our analysis was similar to the entire ARIES dataset. The largest differences were observed for maternal education at birth (with the mothers of included individuals having slightly higher educational attainment) and ethnicity (with the proportion of individuals of white ethnic background being slightly higher in the included individuals). Previous analyses indicated that ARIES is reasonably representative of the entire ALSPAC cohort [28]. At the other timepoints the sample size differed slightly: there were 640 (birth) and 709 (age 15–17) participants.

### 3.2. Association of Breastfeeding with Single CpG Sites

**Figure 1:**
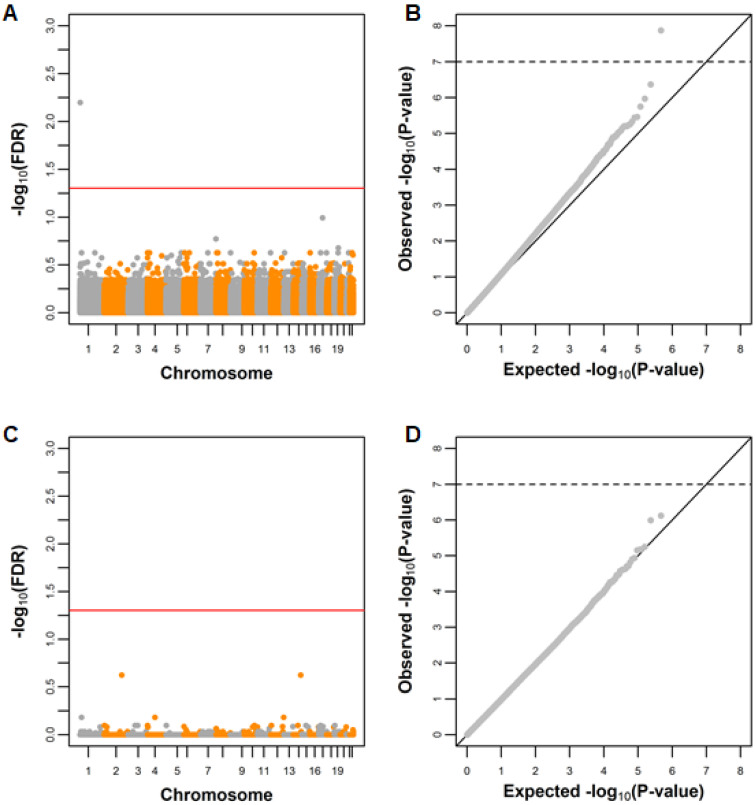
Manhattan (**A**,**C**) and Q-Q plots (**B**,**D**) of the breastfeeding EWAS at age 7. In (**A**,**B**), methylation was compared between never vs. ever breastfed individuals; in (**C**,**D**) methylation was compared according to breastfeeding duration (in categories, assuming a linear trend). Both models were adjusted for cell composition, batch, child genetic ancestry (two principal components), mother’s education, parity, age at birth, folic acid supplementation, maternal smoking, gestational age, type of delivery, birthweight, child sex and age at blood collection.

Figure 1 provides an overall view of the EWAS results. There was no strong indication of genome-wide inflation for breastfeeding analysed in duration categories, assuming a linear trend (genomic inflation factor of 0.97), but there was some indication for the “ever breastfeeding” variable (genomic inflation factor of 1.10). Importantly, the bulk of the distribution closely resembled the expected under the null, with the deviation occurring in the right tail of the distribution of *p*-values. This may be due to breastfeeding having small effects on DNA methylation (in which case detection would require larger samples) in many regions of the genome, rather than due to the presence of systematic bias in the results.

Regarding ever breastfeeding (Table 2), one CpG (cg11414913) achieved FDR < 0.05, and there was suggestive evidence of association for six additional sites (cg00234095, cg04722177, cg03945777, cg17052885, cg05800082 and cg24134845; see Table S6 for a description of those CpGs). None of the sites were located in array probes with potential to cross-hybridize to multiple genomic regions according to Naeem et al. [50]. The results for breastfeeding coded as a categorical variable in duration categories (assuming a linear trend) were null, with no CpGs achieving even suggestive levels of association.

Table 2 shows that methylation in the cg11414913 CpG was 3.2 percent points lower (*p* = 5.2 × 10^−8^) in ever breastfed children. There was also suggestive evidence for an association between breastfeeding and lower methylation in the cg00234095 (β = −1.7; *p* = 4.9 × 10^−7^), cg04722177 (β = −2.9; *p* = 2.7 × 10^−6^), and cg03945777 (β = −0.8; *p* = 3.2 × 10^−6^) sites, and for higher methylation in the cg17052885 (β = 1.8; *p* = 4.9 × 10^−6^), cg05800082 (β = 1.1; *p* = 5.8 × 10^−6^), and cg24134845 (β = 0.2; *p* = 3.3 × 10^−5^) sites. The evidence of an association was greatly attenuated when breastfeeding was analysed continuously (in months), and the regression coefficients were generally similar among different categories of breastfeeding duration. The sensitivity analysis using breastfeeding duration categories by 2-week increments in the first 3 months of age (Table S2) showed no clear trend in the regression estimates comparing each duration category to no breastfeeding. The introduction of formula in the first 3 months did not seem to alter the association of breastfeeding with DNA methylation, as shown in Table S3. Further adjustment for lipid profiles did not substantially influence the beta estimates of the association between breastfeeding and DNA methylation at age 7 (Table S4). Furthermore, DNA methylation at the suggestive CpGs was not associated with gestational age methylation score (Table S5). Altogether, these results indicate that the association between breastfeeding and peripheral blood DNA methylation is unlikely to follow a dose-response relationship but presents a threshold (ever vs. never) pattern.

Table 3 displays the association between ever breastfeeding and peripheral blood methylation at different ages in the CpGs identified in the EWAS. The cg11414913 CpG presented a persistent, directionally-consistent association with breastfeeding at the age of 15–17 years (β = −2.8; *p* = 0.004), and no strong evidence of association at birth (β = −0.4; *p* = 0.631). The cg05800082 CpG presented a similar pattern, although the point estimate was attenuated compared to age 7 years, and presented rather weak statistical evidence of association at the age of 15–17 years (β = 0.6; *p* = 0.083). However, it was reassuring that its point estimate at birth (β = −0.5; *p* = 0.144) was directionally inconsistent with the results at later ages. The CpGs cg00234095, cg03945777 and cg24134845 presented evidence of association at age 7, but neither at birth nor at age 15–17, suggesting a true association with breastfeeding that does not persist until the ages of 15–17. DNA methylation at birth in the two remaining CpGs was associated with breastfeeding in the same direction as the association at the age of 7, suggesting that those associations are substantially influenced by some unaccounted bias source (e.g., unmeasured confounders).

### 3.3. Association between Breastfeeding and Methylation Regions

Given that the QQ-plots were suggestive of small effects of breastfeeding on DNA methylation in many regions of the genome, we complemented the ever breastfeeding EWAS with a search for differentially methylated regions (DMRs)—i.e., two or more CpGs enriched for low *p*-values of the association with breastfeeding (see the Methods for details). In total, 12 DMRs were identified at age 7 (Table 4 and Table S7). There was no strong indication that the association of breastfeeding with different CpGs in the same DMR was generally directionally consistent (Table 4).

When we checked the stability of the associations over time, four DMRs presented evidence of concordance between 7 and 15–17 years, but not between methylation at birth and at age 7 (chromosome:position): 18:106,178–106,850, 9:91296–92146, 22:255,590–256,045, and 8:409,905–410,098 (Table 5). For two DMRs (5:97,867–98,797 and 1:425,524–426,297), there was evidence for directional concordance between birth and 7 years of age, suggesting that the associations between breastfeeding and methylation at age 7 in the CpGs in those DMRs may be distorted by pre-natal confounders. For the remaining six DMRs, there was no evidence for directional concordance between any of the two comparisons, suggesting that the association between breastfeeding and methylation at age 7 in the CpGs in those DMRs may be transient (i.e., childhood specific) or false positives. A sensitivity analysis considering only those CpGs that achieved *p* < 0.05 in at least one time point corroborated the strongest directional consistency between 7 and 15–17 years observed for the four aforementioned DMRs, except the 8:409,905–410,098; importantly, this analysis involved only three CpGs for this DMR (Table S8). Moreover, a fifth DMR—9:365,914–366,989—was identified in this analysis, suggesting that CpGs with weak associations could have diluted the association in the analysis considering all CpGs in the DMR.

## 4. Discussion

In this epigenome-wide association study, having ever been breastfed was associated with peripheral blood methylation in the cg11414913 CpG at ages 7 and 15–17 years, but not at birth. There was suggestive evidence of association between ever been breastfed and age 7 methylation in six additional CpGs, with one—the cg05800082 CpG—also presenting a directionally consistent (although attenuated) point estimate at age 15, but not at birth. Moreover, 12 DMRs were identified at age 7, and three of them presented evidence of directional concordance between ages 7 and 15–17, but not between birth and age 7, in all sensitivity analyses. Our QQ-plots indicated that the associational effect estimates between ever breastfeeding and peripheral blood DNA methylation are generally small. Our analyses did not support a dose-response relationship between breastfeeding and peripheral blood DNA methylation, but were consistent with an effect that depends on whether or not the child was ever breastfed.

The epidemiological literature on breastfeeding and health focuses on well-established effects against infectious diseases, as well as putative long-term effects on obesity, diabetes and cognitive development, among other outcomes [1]. In the present analyses, only one site where methylation differences were detected could be involved in the above traits. Specifically, an online search into the biological role of the genes whose methylation was associated with breastfeeding (see Table S6) showed an effect on the dystonin (DST) gene, which is expressed in the brain and other tissues (www.gtextportal.org, accessed on 2 February 2019). In neural cells, DST (also known as BPAG1) isoforms act as cytoskeletal linker proteins that anchor neural intermediate filaments to the actin cytoskeleton [51]. Alterations in these neuronal functions could link breastfeeding to its putative benefits on cognitive development, although this remains speculative. Other sites affected were not located on genes with known function or were in genes expressed in other tissues such as testis. This may be due to analysing a surrogate tissue, limited statistical power to detect more CpGs, and limited knowledge about the health effects of the methylation sites that were detected. cg11414913, which showed the most robust evidence of association with breastfeeding, is located in an intergenic region with seemingly regulatory properties, as indicated by a conserved region hypersensitive to DNAses. Its nearest gene encodes for Tetratricopeptide Repeat Protein 34 (TTC34), which is overexpressed in the testis. However, it is unclear if there is indeed any relationship between these biological features of cg11414913 and the effects of breastfeeding, an issue that thus requires further investigation. Moreover, the effects of breastfeeding on health and development may be mediated through other epigenetic processes, such as non-coding RNAs [52,53], as well as a host of mechanisms other than epigenetics, including provision of nutrients (e.g., pre-formed long-chain polyunsaturated fatty acids, which is a plausible mediator of the benefits on IQ [54]), antibodies and other immunoactive compounds, antimicrobials, and important effects on the gut microbiome [1].

One of the strengths of this study is that longitudinal measures of DNA methylation allowed not only identifying regions of the methylome associated with breastfeeding, but also assessing if those associations persist until adolescence. Dense phenotyping and genotyping of study participants allowed controlling for several covariates, which were selected using a conceptual model defined a priori. Moreover, DNA methylation data at birth was used as an attempt to identify associations likely driven by residual confounding due to pre-natal factors. To unravel the possibility of residual confounding by maternal smoking, we checked the overlap between the suggestive CpG sites from our breastfeeding EWAS and the largest maternal smoking EWAS [55], and found that none of the sites were amongst the 6073 sites that were associated with maternal smoking during pregnancy, making it less likely that the associations were driven by residual confounding by maternal smoking. However, residual confounding cannot be discounted due to missing confounders (including post-birth factors that may affect breastfeeding quality and duration) measurement error and model misspecification [56]. Therefore, triangulating our findings with those from future studies using designs prone to different potential sources of bias will be important to disentangle causality [57].

In addition to the possibility of residual confounding, another weakness of our study is that our exposure variable was ill-defined. Due to sample size constraints and limitations of self-reported data, it was not possible to use more refined definitions of breastfeeding. Indeed, our main results were related to the binary categorisation, which includes, in the “breastfed” group, highly heterogeneous individuals regarding breastfeeding quality, duration, type of foods given concurrently with breastmilk (for individuals that were non-exclusively breastfed) and after weaning, among other factors. Similarly, the “non-breastfed” group potentially includes individuals that received many different types of foods. This heterogeneity is likely to influence the results in ways that are rather difficult to predict and limits the external validity of our findings.

With regards to the analysis on breastfeeding duration, we opted for a categorisation into groups rather than the continuous breastfeeding variable because the latter is likely prone to substantial measurement error and digit preference in the self-reported months of duration. Moreover, assuming a linear effect over the entire range of breastfeeding duration (which entails assuming, for example that the effect of changing from 0 to 1 month is the same as the effect of changing from 15 to 16 months) seems less plausible than a linear trend over duration categories (which entails assuming, for example, that the effect of changing from 0–3 to 3.01–6 months is the same as the effect of changing from 6.01–12 to >12 months). However, this categorisation did not show evidence of strong associations.

It should also be noted that our study was restricted to peripheral blood. As we discussed elsewhere [22], DNA methylation in blood is unlikely to be a good proxy of DNA methylation in other tissues, such as the brain [58,59,60], thus limiting the capacity of any breastfeeding EWAS using peripheral blood to inform DNA methylation patterns in the target tissue [15,61]—in this example, when assessing if the association between breastfeeding and IQ has a component related to methylation. This may also limit the capacity to identify true signals. However, DNA methylation studies in surrogate tissues are important. These are frequently the only viable alternative in large epidemiological studies, also being able to provide useful information on the range of potential effects of the exposure of interest on DNA methylation, which may then guide future, specific studies such as in vitro studies in cells and in vivo studies in animal models [22].

Another important limitation is that we did not perform a formal replication of our results. However, it is noteworthy that some hits (both in the CpG and DMR analysis) at age 7 years did not present evidence of association at age 15–17 years. This indicates that inflation of type-I error due to multiple-testing was not sufficient for a hit in one age to also present evidence of association in other ages (otherwise, all hits at age 7 years would have also presented evidence of association at age 15–17 years). Therefore, CpGs and DMRs that presented evidence of persistent associations are less likely to be a sole product of multiple testing. However, this reasoning is less clear for transient associations, which could be truly transient effects or merely false positives that do not carry over to adolescence. Although persistent associations are likely to be more robust from a methodological perspective in our study, this does not mean that transient effects are irrelevant. For example, they could trigger the actual processes related to long-term effects (e.g., influences on brain development and IQ in adulthood). Moreover, in our context transient effects mean that associations observed at the age of 7 years did not persist until adolescence, but associations at age 7 would already be persistent effects of breastfeeding. Finally, it is important to consider the loss of individuals to missing data. About 30% of ARIES data were removed due to missing exposure, covariate or outcome data, which reduces the power to find CpG sites related to breastfeeding. Methods for multiple imputation in methylation data [62] are at an early stage and therefore were not used here, but in future these methods will be crucial to maximise the power of an EWAS.

## 5. Conclusions

This study provides important insights into the magnitude and persistence of the association between breastfeeding and peripheral blood DNA methylation. Rather than providing definitive answers on their own, our results will serve to motivate future studies using different designs to improve causal inference, as well as consortium-based efforts—examples of which are already available in the epigenetic epidemiology literature [55,63]—to achieve sample sizes large enough to both improve power and allow replication. Such future efforts will complement and expand our findings by providing robust evidence on the potential effects of breastfeeding on DNA methylation, which may contribute to understand the biological basis of long-term associations between breastfeeding and health and human capital outcomes, and potentially also reveal new biological aspects of breastfeeding.

## Figures and Tables

**Table 1:** Description of the individuals included in the main analysis (ARIES participants with age 7 methylation data and all covariates).

Variable	Statistic/Category ^a^	All ARIES Participants (N = 995)	Participants with Methylation at Age 7 (N = 702)
Maternal education	CSE	8.9%	7.2%
at birth	Vocational education	7.4%	6.0%
	GCE Ordinary level	34.3%	33.8%
	GCE Advanced level	29.1%	29.9%
	Degree	20.3%	23.1%
Maternal age at birth (years)	Mean (SD)	29.5 (4.4)	30.0 (4.4)
Parity	0	46.5%	45.7%
	1	36.9%	37.5%
	2	12.7%	13.4%
	≥3	3.9%	3.4%
Maternal smoking	Never	86.3%	87.7%
in relation to	Before	3.7%	4.0%
pregnancy	During	10.0%	8.3%
Folic acid	No	75.9%	75.9%
supplementation	Yes	24.1%	24.1%
Caesarean section	No	90.4%	90.2%
	Yes	9.6%	9.8%
Birthweight (g)	Mean (SD)	3487 (486)	3490 (476)
Sex	Male	48.9%	49.1%
	Female	51.1%	50.9%
Ethnicity	European	97.0%	99.9%
	Other	3.0%	0.1%
Breastfeeding duration	0	11.1%	10.4%
(months)	0.1–3	32.0%	31.0%
	3.1–6	16.2%	16.2%
	6.1–12	27.6%	28.2%
	>12	13.1%	14.2%

^a^ Mean and SD for continuous variables, and each category (for which proportions are shown) for categorical variables. CSE: Certificate of Secondary Education. GCE: General Certificate of Education. SD: standard deviation.

**Table 2:** Average percent point differences (β) in DNA methylation at age 7 (N = 640) according to breastfeeding.

Breastfeeding	Statistic	CpG						
		cg11414913	cg00234095	cg04722177	cg03945777	cg17052885	cg05800082	cg24134845
Binary (ever vs. never)	*p*-value	5.2 × 10^−8^	4.9 × 10^−7^	2.7 × 10^−6^	3.2 × 10^−6^	4.9 × 10^−6^	5.8 × 10^−6^	3.3 × 10^−5^
	β (SE)	−3.19 (0.59)	−1.74 (0.35)	−2.90 (0.62)	−0.84 (0.18)	1.79 (0.39)	1.05 (0.23)	0.23 (0.06)
0 (reference)	*p*-value	-	-	-	-	-	-	-
	β (SE)	-	-	-	-	-	-	-
0.01–3 months	*p*-value	1.5 × 10^−6^	1.2 × 10^−7^	5.3 × 10^−4^	2.9 × 10^−5^	8.2 × 10^−6^	1.7 × 10^−6^	6.8 × 10^−5^
	β (SE)	−3.19 (0.66)	−2.02 (0.38)	−2.45 (0.71)	−0.85 (0.20)	1.85 (0.41)	1.19 (0.25)	0.25 (0.06)
3.01–6 months	*p*-value	5.4 × 10^−7^	3.3 × 10^−5^	5.8 × 10^−5^	0.005	6.8 × 10^−5^	6.4 × 10^−4^	0.011
	β (SE)	−3.50 (0.70)	−1.88 (0.45)	−3.22 (0.80)	−0.66 (0.23)	1.85 (0.47)	0.94 (0.28)	0.17 (0.07)
6.01–12 months	*p*-value	2.5 × 10^−5^	3.2 × 10^−4^	5.9 × 10^−5^	7.4 × 10^−5^	6.1 × 10^−6^	0.001	2.2 × 10^−4^
	β (SE)	−3.00 (0.71)	−1.59 (0.44)	−3.05 (0.76)	−0.90 (0.23)	2.02 (0.45)	0.87 (0.27)	0.24 (0.06)
>12 months	*p*-value	5.8 × 10^−4^	0.037	1.1 × 10^−6^	1.2 × 10^−4^	0.008	0.001	4.4 × 10^−4^
	β (SE)	−2.96 (0.86)	−0.93 (0.44)	−3.79 (0.78)	−0.99 (0.26)	1.29 (0.49)	1.04 (0.31)	0.25 (0.07)
Linear trend of categories	*p*-value	0.036	0.832	1.7 × 10^−4^	0.007	0.067	0.230	0.020
	β (SE)	−0.42 (0.20)	−0.02 (0.11)	−0.70 (0.19)	−0.16 (0.06)	0.19 (0.10)	0.08 (0.07)	0.04 (0.02)
Continuous (in months)	*p*-value	0.080	0.766	2.5 × 10^−4^	0.035	0.966	0.399	0.289
	β (SE)	−0.09 (0.05)	0.01 (0.03)	−0.18 (0.05)	−0.03 (0.02)	0.00 (0.03)	0.01 (0.02)	0.00 (0.00)

SE: standard error.

**Table 3:** Average percent point differences (β) in DNA methylation at different ages according to breastfeeding.

CpG	Time Point	β	SE	*p*-Value
cg11414913	At birth (N = 702)	−0.44	0.91	0.631
	7 years (N = 640)	−3.19	0.59	5.2 × 10^−8^
	15–17 years (N = 709)	−2.47	0.85	0.004
cg00234095	At birth (N = 702)	0.59	0.57	0.296
	7 years (N = 640)	−1.74	0.35	4.9 × 10^−7^
	15–17 years (N = 709)	0.29	0.43	0.505
cg04722177	At birth (N = 702)	−1.50	0.70	0.032
	7 years (N = 640)	−2.90	0.62	2.7 × 10^−6^
	15–17 years (N = 709)	−1.05	0.78	0.180
cg03945777	At birth (N = 702)	0.42	0.3	0.158
	7 years (N = 640)	−0.84	0.18	3.2 × 10^−6^
	15–17 years (N = 709)	0.10	0.29	0.742
cg17052885	At birth (N = 702)	1.32	0.57	0.022
	7 years (N = 640)	1.79	0.39	4.9 × 10^−6^
	15–17 years (N = 709)	−0.29	0.47	0.547
cg05800082	At birth (N = 702)	−0.53	0.36	0.144
	7 years (N = 640)	1.05	0.23	5.8 × 10^−6^
	15–17 years (N = 709)	0.56	0.32	0.083
cg24134845	At birth (N = 702)	0.04	0.07	0.535
	7 years (N = 640)	0.23	0.06	3.3 × 10^−5^
	15–17 years (N = 709)	0.00	0.08	0.991

SE: standard error.

**Table 4:** Association between peripheral blood DNA methylation at different ages at each DMR and ever breastfeeding.

DMR ^a^			At birth			7 Years			15–17 Years		
Chr	Start	End	β	SE	*p*-Value	β	SE	*p*-Value	β	SE	*p*-Value
5	97,867	98,797	0.30	0.21	0.146	0.43	0.21	0.043	0.30	0.21	0.158
19	365,914	366,989	−0.01	0.34	0.975	0.05	0.34	0.881	−0.04	0.35	0.897
18	106,178	106,850	−0.08	0.77	0.913	0.14	0.75	0.855	0.23	0.77	0.767
1	425,524	426,297	0.26	0.62	0.673	0.33	0.61	0.590	0.16	0.62	0.800
9	91,296	92,146	−0.10	0.33	0.759	−0.18	0.33	0.578	−0.10	0.34	0.755
17	222,498	222,991	−0.01	0.37	0.983	0.00	0.36	0.994	−0.04	0.36	0.913
4	136,643	137,027	−0.03	0.41	0.951	−0.37	0.38	0.324	−0.31	0.41	0.448
22	255,590	256,045	0.40	0.71	0.577	1.18	0.70	0.095	1.06	0.71	0.136
4	33,482	33,808	0.13	2.05	0.950	0.06	2.00	0.978	0.08	2.04	0.967
8	409,905	410,098	0.82	1.31	0.530	1.05	1.32	0.425	1.04	1.32	0.433
1	224,191	225,190	0.03	0.45	0.940	−0.03	0.44	0.951	−0.03	0.45	0.948
9	61,093	61,964	−0.39	0.50	0.432	−0.44	0.49	0.369	−0.39	0.50	0.435

Regression coefficients (β) are average percent point differences in DNA methylation averaged across CpGs that belong to the DMR. P-values are computed for the change in methylation across all CpGs within a DMR. This analysis was performed using linear mixed models to account for the correlation between CpGs in the same DMR. ^a^ Human Genome Assembly GRCh37. Chr: Chromosome. DMR: differentially methylated region. SE: standard error.

**Table 5:** Directional concordance between time points for each individual CpG belonging to the same DMR.

DMR ^a^			Number	At Birth and 7 Years		7 Years and 15–17 Years	
Chr	Start	End	of CpGs	Concordance	*p*-Value	Concordance	*p*-Value
5	97,867	98,797	275	66.2	8.7 × 10^−8^	69.1	2.2 × 10^−10^
19	365,914	366,989	205	47.8	0.576	54.1	0.264
18	106,178	106,850	18	72.2	0.096	83.3	0.008
1	425,524	426,297	64	68.8	0.004	56.3	0.382
9	91,296	92,146	185	54.1	0.303	58.4	0.027
17	222,498	222,991	140	55.7	0.205	49.3	0.933
4	136,643	137,027	13	69.2	0.267	61.5	0.581
22	255,590	256,045	30	63.3	0.200	83.3	3.3 × 10^−4^
4	33,482	33,808	5	60.0	0.999	60.0	0.999
8	409,905	410,098	7	85.7	0.125	100.0	0.016
1	224,191	225,190	129	57.4	0.113	47.3	0.597
9	61,093	61,964	91	57.1	0.208	56.0	0.294

Concordance is shown in %. The analyses were performed using a sign test. ^a^ Human Genome Assembly GRCh37. Chr: Chromosome. DMR: differentially methylated region.

## Supplementary Materials

The following are available online at https://www.mdpi.com/2072-6643/12/11/3309/s1, Table S1. Description of the individuals included in the main analysis, restricting to those with age 7 methylation data available, cord blood methylation and age 15–17 methylation; Table S2. Change in DNA methylation at age 7 according to breastfeeding duration (two-week categories) in the first 3 months at the suggestive sites; Table S3. Change in DNA methylation at age 7 according to introduction of formula in the first 3 months; Table S4. Change in methylation at age 7 according to breastfeeding (ever vs. never) with adjustment for all covariates + serum lipid profiles (total cholesterol, VLDL-cholesterol, HDL-cholesterol, LDL-cholesterol, total triglycerides) in children at age 7 or in the mothers during pregnancy; Table S5. Change in DNA methylation at age 7 per 10 days increase in gestational age at the suggestive sites; Table S6. Description of the CpGs that presented at least suggestive evidence of association with ever breastfeeding in the fully-adjusted analysis at age 7; Table S7. Differentially methylated regions (DMR) in peripheral blood at age 7 according to ever breastfeeding; Table S8. Directional concordance (in %) between time points for each individual CpG belonging to the same differentially methylated region (DMR). Only CpGs that achieved *p* < 0.05 in at least one time point were considered.

## References

[1] Victora C.G., Bahl R., Barros A.J.D., França G.V.A., Horton S., Krasevec J., Murch S., Sankar M.J., Walker N., Rollins N.C. (2016). Breastfeeding in the 21st century: Epidemiology, mechanisms, and lifelong effect. Lancet.

[2] Horta B.L., Loret De Mola C., Victora C.G. (2015). Long-term consequences of breastfeeding on cholesterol, obesity, systolic blood pressure and type 2 diabetes: A systematic review and meta-analysis. Acta Paediatr. Int. J. Paediatr..

[3] Brion M.J.A., Lawlor D.A., Matijasevich A., Horta B., Anselmi L., Araújo C.L., Menezes A.M.B., Victora C.G., Davey Smith G. (2011). What are the causal effects of breastfeeding on IQ, obesity and blood pressure? Evidence from comparing high-income with middle-income cohorts. Int. J. Epidemiol..

[4] Kramer M.S., Aboud F., Mironova E., Vanilovich I., Platt R.W., Matush L., Igumnov S., Fombonne E., Bogdanovich N., Ducruet T. (2008). Breastfeeding and child cognitive development: New evidence from a large randomized trial. Arch. Gen. Psychiatry.

[5] Horta B.L., Loret De Mola C., Victora C.G. (2015). Breastfeeding and intelligence: A systematic review and meta-analysis. Acta Paediatr. Int. J. Paediatr..

[6] Martin R.M., Patel R., Kramer M.S., Guthrie L., Vilchuck K., Bogdanovich N., Sergeichick N., Gusina N., Foo Y., Palmer T. (2013). Effects of promoting longer-term and exclusive breastfeeding on adiposity and insulin-like growth factor-I at age 11.5 years: A randomized trial. JAMA J. Am. Med. Assoc..

[7] Martin R.M., Kramer M.S., Patel R., Rifas-Shiman S.L., Thompson J., Yang S., Vilchuck K., Bogdanovich N., Hameza M., Tilling K. (2017). Effects of promoting long-term, exclusive breastfeeding on adolescent adiposity, blood pressure, and growth trajectories: A secondary analysis of a randomized clinical trial. JAMA Pediatr..

[8] Martin R.M., Patel R., Kramer M.S., Vilchuck K., Bogdanovich N., Sergeichick N., Gusina N., Foo Y., Palmer T., Thompson J. (2014). Effects of promoting longer-term and exclusive breastfeeding on cardiometabolic risk factors at age 11.5 years: A cluster-randomized, controlled trial. Circulation.

[9] Yang S., Martin R.M., Oken E., Hameza M., Doniger G., Amit S., Patel R., Thompson J., Rifas-Shiman S.L., Vilchuck K. (2018). Breastfeeding during infancy and neurocognitive function in adolescence: 16-year follow-up of the PROBIT cluster-randomized trial. PLoS Med..

[10] Relton C.L., Hartwig F.P., Davey Smith G. (2015). From stem cells to the law courts: DNA methylation, the forensic epigenome and the possibility of a biosocial archive. Int. J. Epidemiol..

[11] Richmond R.C., Simpkin A.J., Woodward G., Gaunt T.R., Lyttleton O., McArdle W.L., Ring S.M., Smith A.D.A.C., Timpson N.J., Tilling K. (2015). Prenatal exposure to maternal smoking and offspring DNA methylation across the lifecourse: Findings from the Avon Longitudinal Study of Parents and Children (ALSPAC). Hum. Mol. Genet..

[12] Rakyan V.K., Down T.A., Balding D.J., Beck S. (2011). Epigenome-wide association studies for common human diseases. Nat. Rev. Genet..

[13] Kiefer J.C. (2007). Epigenetics in development. Dev. Dyn..

[14] Huang K., Fan G. (2010). DNA methylation in cell differentiation and reprogramming: An emerging systematic view. Regen. Med..

[15] Relton C.L., Davey Smith G. (2010). Epigenetic epidemiology of common complex disease: Prospects for prediction, prevention, and treatment. PLoS Med..

[16] Kaelin W.G., McKnight S.L. (2013). Influence of metabolism on epigenetics and disease. Cell.

[17] Tobi E.W., Slieker R.C., Luijk R., Dekkers K.F., Stein A.D., Xu K.M., Slagboom P.E., Van Zwet E.W., Lumey L.H., Heijmans B.T. (2018). DNA methylation as a mediator of the association between prenatal adversity and risk factors for metabolic disease in adulthood. Sci. Adv..

[18] Birney E., Davey Smith G., Greally J.M. (2016). Epigenome-wide Association Studies and the Interpretation of Disease -Omics. PLoS Genet..

[19] Richmond R., Relton C., Davey Smith G. (2018). What evidence is required to suggest that DNA methylation mediates the association between prenatal famine exposure and adulthood disease?. Sci. Adv..

[20] Verduci E., Banderali G., Barberi S., Radaelli G., Lops A., Betti F., Riva E., Giovannini M. (2014). Epigenetic effects of human breast milk. Nutrients.

[21] Mischke M., Plösch T. (2013). More than just a gut instinct-the potential interplay between a baby’s nutrition, its gut microbiome, and the epigenome. Am. J. Physiol. Regul. Integr. Comp. Physiol..

[22] Hartwig F.P., De Mola C.L., Davies N.M., Victora C.G., Relton C.L. (2017). Breastfeeding effects on DNA methylation in the offspring: A systematic literature review. PLoS ONE.

[23] Naumova O.Y., Odintsova V.V., Arincina I.A., Rychkov S.Y., Muhamedrahimov R.J., Shneider Y.V., Grosheva A.N., Zhukova O.V., Grigorenko E.L. (2019). A Study of the Association between Breastfeeding and DNA Methylation in Peripheral Blood Cells of Infants. Russ. J. Genet..

[24] Sherwood W.B., Bion V., Lockett G.A., Ziyab A.H., Soto-Ramírez N., Mukherjee N., Kurukulaaratchy R.J., Ewart S., Zhang H., Arshad S.H. (2019). Duration of breastfeeding is associated with leptin (LEP) DNA methylation profiles and BMI in 10-year-old children. Clin. Epigenetics.

[25] Pauwels S., Symons L., Vanautgaerden E.-L., Ghosh M., Duca R.C., Bekaert B., Freson K., Huybrechts I., Langie S.A.S., Koppen G. (2019). The influence of the duration of breastfeeding on the infant’s metabolic epigenome. Nutrients.

[26] Odintsova V.V., Hagenbeek F.A., Suderman M., Caramaschi D., Van Beijsterveldt C.E.M., Kallsen N.A., Ehli E.A., Davies G.E., Sukhikh G.T., Fanos V. (2019). DNA methylation signatures of breastfeeding in buccal cells collected in mid-childhood. Nutrients.

[27] Sherwood W.B., Kothalawala D.M., Kadalayil K., Ewart S., Zhang H., Karmaus W., Arshad S.H., Holloway J.W., Rezwan F.I. (2020). Epigenome-Wide Association Study Reveals Duration of Breastfeeding Is Associated with Epigenetic Differences in Children. Int. J. Environ. Res. Public Health.

[28] Relton C.L., Gaunt T., McArdle W., Ho K., Duggirala A., Shihab H., Woodward G., Lyttleton O., Evans D.M., Reik W. (2015). Data resource profile: Accessible resource for integrated epigenomic studies (ARIES). Int. J. Epidemiol..

[29] Golding G., Pembrey P., Jones J. (2001). ALSPAC—The Avon Longitudinal Study of Parents and Children I. Study methodology. Paediatr. Perinat. Epidemiol..

[30] Boyd A., Golding J., Macleod J., Lawlor D.A., Fraser A., Henderson J., Molloy L., Ness A., Ring S., Davey Smith G. (2013). Cohort profile: The ’Children of the 90s’-The index offspring of the avon longitudinal study of parents and children. Int. J. Epidemiol..

[31] Fraser A., Macdonald-wallis C., Tilling K., Boyd A., Golding J., Davey Smith G., Henderson J., Macleod J., Molloy L., Ness A. (2013). Cohort profile: The avon longitudinal study of parents and children: ALSPAC mothers cohort. Int. J. Epidemiol..

[32] Touleimat N., Tost J. (2012). Complete pipeline for Infinium^®^ Human Methylation 450K BeadChip data processing using subset quantile normalization for accurate DNA methylation estimation. Epigenomics.

[33] Pidsley R., Wong C.C.Y., Volta M., Lunnon K., Mill J., Schalkwyk L.C. (2013). A data-driven approach to preprocessing Illumina 450K methylation array data. BMC Genom..

[34] Min J.L., Hemani G., Davey Smith G., Relton C., Suderman M. (2018). Meffil: Efficient normalization and analysis of very large DNA methylation datasets. Bioinformatics.

[35] Du P., Zhang X., Huang C.C., Jafari N., Kibbe W.A., Hou L., Lin S.M. (2010). Comparison of Beta-value and M-value methods for quantifying methylation levels by microarray analysis. BMC Bioinform..

[36] Price A.L., Patterson N.J., Plenge R.M., Weinblatt M.E., Shadick N.A., Reich D. (2006). Principal components analysis corrects for stratification in genome-wide association studies. Nat. Genet..

[37] Bakulski K.M., Feinberg J.I., Andrews S.V., Yang J., Brown S., McKenney S.L., Witter F., Walston J., Feinberg A.P., Fallin M.D. (2016). DNA methylation of cord blood cell types: Applications for mixed cell birth studies. Epigenetics.

[38] Houseman E.A., Accomando W.P., Koestler D.C., Christensen B.C., Marsit C.J., Nelson H.H., Wiencke J.K., Kelsey K.T. (2012). DNA methylation arrays as surrogate measures of cell mixture distribution. BMC Bioinform..

[39] Leek J.T., Storey J.D. (2007). Capturing heterogeneity in gene expression studies by surrogate variable analysis. PLoS Genet..

[40] Benjamini Y., Hochberg Y. (1995). Controlling the False Discovery Rate: A Practical and Powerful Approach to Multiple Testing. J. R. Stat. Soc. Ser. B.

[41] Würtz P., Kangas A.J., Soininen P., Lawlor D.A., Davey Smith G., Ala-Korpela M. (2017). Quantitative Serum Nuclear Magnetic Resonance Metabolomics in Large-Scale Epidemiology: A Primer on -Omic Technologies. Am. J. Epidemiol..

[42] Bohlin J., Håberg S.E., Magnus P., Reese S.E., Gjessing H.K., Magnus M.C., Parr C.L., Page C.M., London S.J., Nystad W. (2016). Prediction of gestational age based on genome-wide differentially methylated regions. Genome Biol..

[43] Simpkin A.J., Suderman M., Howe L.D. (2017). Epigenetic clocks for gestational age: Statistical and study design considerations. Clin. Epigenetics.

[44] Davey Smith G. (2008). Assessing intrauterine influences on offspring health outcomes: Can epidemiological studies yield robust findings?. Basic Clin. Pharmacol. Toxicol..

[45] Pedersen B.S., Schwartz D.A., Yang I.V., Kechris K.J. (2012). Comb-p: Software for combining, analyzing, grouping and correcting spatially correlated P-values. Bioinformatics.

[46] Jaffe A.E., Murakami P., Lee H., Leek J.T., Fallin M.D., Feinberg A.P., Irizarry R.A. (2012). Bump hunting to identify differentially methylated regions in epigenetic epidemiology studies. Int. J. Epidemiol..

[47] Sharp G.C., Ho K., Davies A., Stergiakouli E., Humphries K., McArdle W., Sandy J., Davey Smith G., Lewis S.J., Relton C.L. (2017). Distinct DNA methylation profiles in subtypes of orofacial cleft. Clin. Epigenetics.

[48] Kechris K.J., Biehs B., Kornberg T.B. (2010). Generalizing moving averages for tiling arrays using combined P-value statistics. Stat. Appl. Genet. Mol. Biol..

[49] Šidák Z. (1967). Rectangular Confidence Regions for the Means of Multivariate Normal Distributions. J. Am. Stat. Assoc..

[50] Naeem H., Wong N.C., Chatterton Z., Hong M.K.H., Pedersen J.S., Corcoran N.M., Hovens C.M., Macintyre G. (2014). Reducing the risk of false discovery enabling identification of biologically significant genome-wide methylation status using the HumanMethylation450 array. BMC Genom..

[51] Leung C.L., Zheng M., Prater S.M., Liem R.K.H. (2001). The BPAG1 locus: Alternative splicing produces multiple isoforms with distinct cytoskeletal linker domains, including predominant isoforms in neurons and muscles. J. Cell Biol..

[52] Karlsson O., Rodosthenous R.S., Jara C., Brennan K.J., Wright R.O., Baccarelli A.A., Wright R.J. (2016). Detection of long non-coding RNAs in human breastmilk extracellular vesicles: Implications for early child development. Epigenetics.

[53] Alsaweed M., Hartmann P.E., Geddes D.T., Kakulas F. (2015). Micrornas in breastmilk and the lactating breast: Potential immunoprotectors and developmental regulators for the infant and the mother. Int. J. Environ. Res. Public Health.

[54] Innis S.M. (2007). Dietary (n-3) fatty acids and brain development. J. Nutr..

[55] Joubert B.R., Felix J.F., Yousefi P., Bakulski K.M., Just A.C., Breton C., Reese S.E., Markunas C.A., Richmond R.C., Xu C.J. (2016). DNA Methylation in Newborns and Maternal Smoking in Pregnancy: Genome-wide Consortium Meta-analysis. Am. J. Hum. Genet..

[56] Phillips A.N., Davey Smith G. (1991). How independent are ‘independent’ effects? relative risk estimation when correlated exposures are measured imprecisely. J. Clin. Epidemiol..

[57] Lawlor D.A., Tilling K., Smith G.D. (2016). Triangulation in aetiological epidemiology. Int. J. Epidemiol..

[58] Davies M.N., Volta M., Pidsley R., Lunnon K., Dixit A., Lovestone S., Coarfa C., Harris R.A., Milosavljevic A., Troakes C. (2012). Functional annotation of the human brain methylome identifies tissue-specific epigenetic variation across brain and blood. Genome Biol..

[59] Walton E., Hass J., Liu J., Roffman J.L., Bernardoni F., Roessner V., Kirsch M., Schackert G., Calhoun V., Ehrlich S. (2016). Correspondence of DNA methylation between blood and brain tissue and its application to schizophrenia research. Schizophr. Bull..

[60] Hannon E., Lunnon K., Schalkwyk L., Mill J. (2015). Interindividual methylomic variation across blood, cortex, and cerebellum: Implications for epigenetic studies of neurological and neuropsychiatric phenotypes. Epigenetics.

[61] Heijmans B.T., Mill J. (2012). Commentary: The seven plagues of epigenetic epidemiology. Int. J. Epidemiol..

[62] Wu C., Demerath E.W., Pankow J.S., Bressler J., Fornage M., Grove M.L., Chen W., Guan W. (2016). Imputation of missing covariate values in epigenome-wide analysis of DNA methylation data. Epigenetics.

[63] Gruzieva O., Xu C.J., Breton C.V., Annesi-Maesano I., Antó J.M., Auffray C., Ballereau S., Bellander T., Bousquet J., Bustamante M. (2017). Epigenome-wide meta-analysis of methylation in children related to prenatal NO2 air pollution exposure. Environ. Health Perspect..

