# Supplementary material for: Association between Breastfeeding and DNA Methylation over the Life Course: Findings from the Avon Longitudinal Study of Parents and Children (ALSPAC)
Source: Nutrients. 2020 Oct 29;12(11):3309. doi: 10.3390/nu12113309 (PMC7692466; doi:10.3390/nu12113309)
Supplement: Supplementary file 1 [file nutrients-12-03309-s001.pdf]

Supplementary Text

Biological characterisation of the CpGs and DMRs

Methods

The nearest gene to each CpG site was extracted from the annotation file provided by Illumina. We used the UCSC Genome Browser (https://genome.ucsc.edu/cgi-bin/hgGateway; GRCh37/hg19 Assembly) where no genes were available, and to identify other biological features – focusing on DNase I hypersensitivity, presence of binding sites of transcription factors, and conservation among vertebrates – of the regions containing the identified CpGs and DMRs. Features of identified genes (and encoded proteins) were extracted from GeneCards®: The Human Gene Database (http://www.genecards.org/) and from GeneEntrez (https://www.ncbi.nlm.nih.gov/gene). Linked diseases were identified using the Online Mendelian Inheritance in Man (OMIM) database (https://www.ncbi.nlm.nih.gov/omim/).

Results

cg11414913, which presented the most robust statistical evidence of association with breastfeeding, is located in an intergenic region, with the nearest gene being the *TTC34* gene. This gene is overexpressed in the testis, but largely unknown regarding its biological roles, although there is some indication of a relation with multiple sclerosis and lung cancer. The region around this CpG is highly conserved among vertebrates and contains a 249 bp region (which includes the CpG) that presents DNase I hypersensitivity (which is related to more transcriptional activity) in six cell/tissue

types, including lung carcinoma, prostate adenocarcinoma and pancreatic islets. cg05800082, which presented some evidence of persistent association with breastfeeding, is located within the *DST* gene, which is expressed in many tissues, including skin and brain. This gene encodes isoforms of cytoskeletal linker proteins that present tissue-specificity regarding expression and function: while some isoforms expressed in epithelial tissues anchor keratin-containing intermediate filaments to hemidesmosomes, other isoforms – mainly expressed in neural and muscle tissue – anchor neural intermediate filaments to the actin cytoskeleton. Mutations in the *DST* gene have also been implicated in neuronal and skin disorders. Moreover, the region spanning this CpG presents DNase I hypersensitivity in 5 cell/tissue types and enrichment of the H3K27Ac histone mark, which is also related to enhanced transcription.

Regarding DMRs, the 18:106,178-106,850 region is located within the *DUX4* gene, which encodes a transcriptional activator of *PITX1*, and is linked to autosomal dominant facioscapulohumeral muscular dystrophy (FSHD). It is expressed in the testis, and in muscle tissues of FSHD patients. The 9:91,296-92,146 region is located 1,719 bp away from the *PGM5P3-AS1* gene, which encodes a non-coding RNA of unknown function. The 22:255,590-25,6045 region did not present any obvious important biological feature in a 100,000 bp window centred at the DMR. Two additional DMRs presented weaker evidence of a persistent association with breastfeeding. One was the 8:409,905-410,098 region located in the *FBXO25* gene, which encodes a protein that is overexpressed in the testis and belong to the family of F-box proteins, which are components of a ubiquitin protein ligase complex. The second was the 19:365,914-

366,989 region located in the *THEG* gene, which encodes a nuclear protein specifically in the nucleus of haploid male germ cells, with a possible role in spermatogenesis.

Supplementary Tables

Table S1. Description of the individuals included in the main analysis, restricting to those with age 7 methylation data available, cord blood methylation and age 15-17 methylation.

Variable	Statistic/ category[a]	Participants cord (N=640)	Participants age 7 (N=702)	Participants age 15-17 (N=709)
Maternal education at birth	CSE	8.00%	7.2%	7.30%
	Vocational education	6.70%	6.0%	6.10%
	GCE O level	32.30%	33.8%	33.70%
	GCE A level	30.20%	29.9%	29.80%
	Degree	22.80%	23.1%	23.10%
Maternal age at birth (years)	Mean (SD)	29.9 (4.4)	30.0 (4.4)	29.9 (4.4)
Parity	0	46.2%	45.7%	46.10%
	1	37.3%	37.5%	37.80%
	2	13%	13.4%	12.80%
	≥3	3.5%	3.4%	3.30%
Maternal smoking in relation to pregnancy	Never	87.8%	87.7%	88.30%
	Before	4.2%	4.0%	3.70%
	During	8%	8.3%	8.00%
Folic acid supplementation	No	75.6%	75.9%	76.70%
	Yes	24.4%	24.1%	23.30%
Caesarean section	No	90.3%	90.2%	90.60%
	Yes	9.70%	9.8%	9.40%
Birthweight (g)	Mean (SD)	3496.4 (470.1)	3490 (476)	3494.6 (478.6)
Sex	Male	48.10%	49.1%	9.50%
	Female	51.90%	50.9%	31.20%
Ethnicity	European	99.80%	99.9%	99.90%
	Other	0.2%	0.1%	0.10%
Breastfeeding duration (months)	0	10.70%	10.4%	9.50%
	0.1-3	30.90%	31.0%	31.20%
	3.1-6	15.50%	16.2%	16.80%
	6.1-12	28.50%	28.2%	28.30%
	>12	14.40%	14.2%	14.20%

[a]Mean and SD for continuous variables, and each category (for which proportions are shown) for categorical variables.
CSE: Certificate of Secondary Education. GCE: General Certificate of Education. O level: Ordinary level. A level: Advanced level. SD: standard deviation.

Table S2. Change in DNA methylation at age 7 according to breastfeeding duration (two-week categories) in the first 3 months at the suggestive sites.

Breastfeeding	N	Statistic	CpG						
			cg11414913	cg00234095	cg04722177	cg03945777	cg17052885	cg05800082	cg24134845
**0 (reference)**	69	P-value	-	-	-	-	-	-	-
		β (SE)	-	-	-	-	-	-	-
**2 weeks**	47	P-value	0.002	0.004	0.056	0.009	5.61×10^{-6}	6.14×10^{-5}	5.25×10^{-4}
		β (SE)	-2.83 (0.94)	-1.83 (0.64)	-1.85 (0.96)	-0.82 (0.31)	2.36 (0.52)	1.47 (0.37)	0.31 (0.09)
**4 weeks**	32	P-value	0.021	0.003	0.551	8.68×10^{-4}	0.038	0.007	0.004
		β (SE)	-2.81 (1.22)	-1.89 (0.65)	-0.76 (1.27)	-1.05 (0.32)	1.63 (0.79)	1.02 (0.38)	0.34 (0.12)
**6 weeks**	13	P-value	0.087	1.36×10^{-4}	0.011	0.715	0.127	0.010	0.272
		β (SE)	-3.39 (1.98)	-2.63 (0.69)	-3.88 (1.53)	-0.19 (0.51)	1.55 (1.01)	1.19 (0.46)	0.14 (0.13)
**8 weeks**	37	P-value	0.003	0.005	2.65×10^{2}	0.022	0.024	9.1×10^{-4}	0.038
		β (SE)	-4.07 (1.36)	-1.80 (0.64)	-4.69 (1.12)	-0.74 (0.32)	1.24 (0.55)	1.28 (0.39)	0.24 (0.11)
**10 weeks**	7	P-value	0.554	0.092	0.629	0.826	5.42×10^{-6}	0.014	0.233
		β (SE)	-1.31 (2.22)	-2.00 (1.19)	-1.24 (2.57)	-0.17 (0.79)	3.46 (0.76)	2.06 (0.84)	0.19 (0.16)
**12 weeks**	466	P-value	7.38×10^{-8}	6.90×10^{-6}	2.82×10^{-7}	7.00×10^{-6}	1.04×10^{-5}	2.03×10^{-5}	1.1×10^{-4}
		β (SE)	-3.29 (0.61)	-1.67 (0.37)	-3.30 (0.64)	-0.86 (0.19)	1.85 (0.42)	1.02 (0.24)	0.22 (0.06)

SE: standard error

Table S3. Change in DNA methylation at age 7 according to introduction of formula in the first 3 months.

Breastfeeding	N	Statistic	CpG						
			**cg11414913**	**cg00234095**	**cg04722177**	**cg03945777**	**cg17052885**	**cg05800082**	**cg24134845**
**No breastfeeding (reference)**	73	P-value	-	-	-	-	-	-	-
		β (SE)	-	-	-	-	-	-	-
**Mixed feeding**	513	P-value	2.32×10^{-6}	1.26×10^{-6}	4.10×10^{-6}	3.39×10^{-6}	1.19×10^{-5}	9.12×10^{-7}	1.6×10^{-4}
		β (SE)	-3.04 (0.64)	-1.65 (0.34)	-2.97 (0.65)	-0.86 (0.18)	1.72 (0.39)	1.12 (0.23)	0.22 (0.06)
**Exclusive breastfeeding**	109	P-value	2.1×10^{-4}	2.74×10^{-5}	8.70×10^{-6}	0.005	7.99×10^{-5}	0.001	0.008
		β (SE)	-2.71 (0.73)	-1.90 (0.45)	-3.63 (0.82)	-0.66 (0.23)	1.94 (0.49)	0.97 (0.30)	0.19 (0.07)

SE: standard error

Table S4. Change in methylation at age 7 according to breastfeeding (ever vs. never) with adjustment for all covariates + serum lipid profiles (total cholesterol, VLDL-cholesterol, HDL-cholesterol, LDL-cholesterol, total triglycerides) in children at age 7 or in the mothers during pregnancy.

**Breastfeeding**	**N**	**Statistic**	**CpG**						
			**cg11414913**	**cg00234095**	**cg04722177**	**cg03945777**	**cg17052885**	**cg05800082**	**cg24134845**
**Ever vs never**	702	P-value	1.83e-07	8.88e-08	3.34e-05	3.79e-05	1.55e-05	1.39e-05	0.000182
**(adjusted for child lipids)**		β (SE)	-3.18 (0.61)	-1.92 (0.36)	-2.79 (0.67)	-0.84 (0.20)	1.75 (0.41)	1.08 (0.25)	0.22 (0.06)
**Ever vs never**	702	P-value	9.27e-05	2.07e-06	4.24e-05	0.00129	0.00375	0.00174	0.00476
**(adjusted for mother's lipids)**		β (SE)	-2.89 (0.74)	-1.88 (0.40)	-3.43 (0.84)	-0.82 (0.25)	1.61 (0.55)	0.96 (0.31)	0.20 (0.07)

VLDL: very low-density lipoproteins
HDL: high-density lipoproteins
LDL: low-density lipoproteins
SE: standard error

Table S5. Change in DNA methylation at age 7 per 10 days increase in gestational age at the suggestive sites.

Breastfeeding	N	Statistic	CpG						
			cg11414913	cg00234095	cg04722177	cg03945777	cg17052885	cg05800082	cg24134845
DNA methylation	625	P-value	0.272	0.653	0.255	0.855	0.565	0.748	0.172
		β (SE)	-0.004 (0.004)	0.002 (0.006)	-0.004 (0.003)	0.0003 (0.002)	0.001 (0.002)	-0.0005 (0.001)	-0.0007 (0.0005)

Table S6. Description of the CpGs that presented at least suggestive evidence of association with ever breastfeeding in the fully-adjusted analysis at age 7.

CpG	Chromosome: position (bp)[a]	Nearest gene	Distance (bp) to nearest gene
cg11414913	1:2,799,662	*TTC34*	93,432
cg00234095	17:39,440,474	*KRTAP9-7*	8,015
cg04722177	19:39,737,768	*IFNL4*	Intragenic
cg03945777	7:157,514,049	*PTPRN2*	Intragenic
cg17052885	17:78,896,012	*RPTOR*	Intragenic
cg05800082	6:56,508,429	*DST*	Intragenic
cg24134845	10:100,992,149	*HPSE2*	Intragenic

[a]Human Genome Assembly GRCh37.
bp: base pairs.

Table S7. Differentially methylated regions (DMR) in peripheral blood at age 7 according to ever breastfeeding.

DMR (Chr:Start-End[a])	Number of CpGs	P-value	Nearest gene	Distance (bp) to nearest gene
5:97,867-98,797	275	3.2×10^{-6}	*PLEKHG4B*	Intragenic
19:365,914-366,989	205	9.7×10^{-5}	*THEG*	Intragenic
18:106,178-106,850	18	0.001	*DUX4*	Intragenic
1:425,524-426,297	64	0.002	*BC036251*	4,458
9:91,296-92,146	185	0.003	*PGM5P3-AS1*	1,719
17:222,498-222,991	140	0.003	*RPH3AL*	19,865
4:136,643-137,027	13	0.007	*ZNF595/ZNF718*	Intragenic
22:255,590-256,045	30	0.012	*AK022914*	15,894,215
4:33,482-33,808	5	0.019	*ZNF595/ZNF718*	19,419
8:409,905-410,098	7	0.025	*FBXO25*	Intragenic
1:224,191-225,190	129	0.045	*LOC729737*	83,625
9:61,093-61,964	91	0.046	*AY343892*	10,734

[a]Human Genome Assembly GRCh37.
[b]No gene within a 100,000 bp window centred at this region.
Chr: chromosome. bp: base pairs.

Table S8. Directional concordance (in %) between time points for each individual CpG belonging to the same differentially methylated region (DMR). Only CpGs that achieved P<0.05 in at least one time point were considered.

DMR	Number	At birth and 7 years		7 years and 15-17 years	
(Chr:Start-End[a])	of CpGs	Concordance	P-value	Concordance	P-value
5:97,867-98,797	69	72.5	2.4×10^{-4}	85.5	1.4×10^{-9}
19:365,914-366,989	38	52.6	0.871	68.4	0.034
18:106,178-106,850	8	75.0	0.289	100.0	0.008
1:425,524-426,297	15	80.0	0.035	73.3	0.118
9:91,296-92,146	38	63.2	0.143	71.1	0.014
17:222,498-222,991	22	45.5	0.832	50.0	0.999
4:136,643-137,027	3	100.0	0.250	66.7	0.999
22:255,590-256,045	16	68.8	0.210	93.8	0.001
4:33,482-33,808	2	50.0	0.999	0.0	0.500
8:409,905-410,098	3	100.0	0.250	100.0	0.250
1:224,191-225,190	23	39.1	0.405	56.5	0.678
9:61,093-61,964	24	75.0	0.023	66.7	0.152

[a]Human Genome Assembly GRCh37.
Chr: Chromosome.
